# Vesnarinone, a differentiation inducing drug, directly activates *p21^waf1^* gene promoter via Sp1 sites in a human salivary gland cancer cell line

**DOI:** 10.1038/sj.bjc.6600592

**Published:** 2002-10-21

**Authors:** F Omotehara, H Kawamata, D Uchida, S Hino, K Nakashiro, T Fujimori

**Affiliations:** Department of Surgical and Molecular Pathology, Dokkyo University School of Medicine, 880 Kitakobayashi, Mibu, Shimo-Tsuga, Tochigi, 321-0293, Japan; Second Department of Oral and Maxillofacial Surgery, Tokushima University School of Dentistry, 3-18-15 Kuramoto, Tokushima, 770-8504, Japan; Division of Genetic Information, Institute for Genome Research, University of Tokushima, 3-18-15 Kuramoto, Tokushima, 770-8503, Japan; Department of Oral and Maxillofacial Surgery, Ehime University School of Medicine, 454 Shitsukawa, Shigenobu, Onsen, Ehime, 791-0295, Japan

**Keywords:** vesnarinone, p21^waf1^, transcription, Sp1, histone-acetylation, salivary gland cancer

## Abstract

We previously demonstrated that a differentiation inducing drug, vesnarinone induced the growth arrest and *p21^waf1^* gene expression in a human salivary gland cancer cell line, TYS. In the present study, we investigated the mechanism of the induction of *p21^waf1^* gene by vesnarinone in TYS cells. We constructed several reporter plasmids containing the *p21^waf1^* promoter, and attempted to identify vesnarinone-responsive elements in the *p21^waf1^* promoter. By the luciferase reporter assay, we identified the minimal vesnarinone-responsive element in the *p21^waf1^* promoter at −124 to −61 relative to the transcription start site. Moreover, we demonstrated by electrophoretic mobility shift assay that Sp1 and Sp3 transcription factors bound to the vesnarinone-responsive element. Furthermore, we found that vesnarinone induced the histone hyperacetylation in TYS cells. These results suggest that vesnarinone directly activates *p21^waf1^* promoter via the activation of Sp1 and Sp3 transcription factors and the histone hyperacetylation in TYS cells.

*British Journal of Cancer* (2002) **87**, 1042–1046. doi:10.1038/sj.bjc.6600592 www.bjcancer.com

© 2002 Cancer Research UK

## 

We have previously demonstrated that a differentiation inducing drug, vesnarinone inhibits the growth of a human salivary gland cancer cell line, TYS, and induces the expression of p21^waf1^, a potent inhibitor of cyclin dependent kinase (Sato *et al*, 1997a; Kawamata *et al*, 1998). Vesnarinone is currently used as a chemotherapeutic agent for head and neck cancer combined with radiation in several countries, such as Japan (Sato *et al*, 1997b,c), the United States and India. *p21^waf1^* is a gene functioning as a cell cycle blocker, and its expression is usually regulated at transcriptional level. p21^waf1^ is known to inhibit cyclin dependent kinase activity in p53-mediated cell cycle arrest induced by DNA damage (El-Deiry *et al*, 1993). Further studies have indicated that *p21^waf1^* is also regulated by other transcription factors during cell differentiation and growth arrest (Dulic *et al*, 1994; Jiang *et al*, 1994). *p21^waf1^* promoter contains not only p53-binding sites but also several transcription factor responsive elements (Datto *et al*, 1995; Nakano *et al*, 1997). One of the responsive elements is for a transcription factor, Sp1. Sp1 responsive elements are located on the upstream of TATA box of *p21^waf1^* promoter. It is reported that several extracellular stimuli including butyrate (Nakano *et al*, 1997), transforming growth factor-β (Datto *et al*, 1995), phorbol esters (Biggs *et al*, 1996), okadaic acid (Biggs *et al*, 1996) and retinoic acid (Liu *et al*, 1996) activate the transcription of *p21^waf1^* gene through the Sp1 responsive elements.

Because TYS cells are reported to have a mutated *p53* gene (Sato *et al*, 1997a), the expression of *p21^waf1^* gene and the growth arrest induced by vesnarinone may be conducted by the p53-independent pathway in TYS cells. In order to use vesnarinone more effectively on the patients with several malignancies, including head and neck cancer, the molecular mechanisms of the growth inhibitory effect of vesnarinone should be studied. In this experiment, we attempted to identify the vesnarinone-responsive elements in the *p21^waf1^* promoter, and clarify the molecular mechanisms of transcriptional activation of *p21^waf1^* gene by treatment with vesnarinone in a human salivary gland cancer cell line, TYS.

## MATERIALS AND METHODS

### Cell culture and reagents

TYS cells (Yanagawa *et al*, 1986) were grown in Dulbecco's modified Eagle medium (DMEM; Life Technologies, Inc., Gaithersburg, MD, USA) supplemented with 10% foetal calf serum (FCS; Bio-Whittaker, Walkersville, MD), 100 μg ml^−1^ streptomycin, 100 U ml^−1^ penicillin (Life Technologies, Inc.), and 0.25 μg ml^−1^ amphotericin B (Life Technologies, Inc.) in a humidified atmosphere of 95% air and 5% CO_2_ at 37°C. Vesnarinone (Otsuka Pharmaceutical Company, Tokyo, Japan) was dissolved in dimethyl sulphoxide (DMSO; Sigma, St. Louis, MO, USA) at a concentration of 10 mg ml^−1^ as the first stock solution, and the first stock solution was diluted with the complete culture medium described above. Trichostatin A (TSA; Wako, Osaka, Japan) was dissolved in ethanol at a concentration of 1 mg ml^−1^, and diluted with the complete culture medium at 10 μg ml^−1^.

### Plasmid preparation

**Figure 1:**
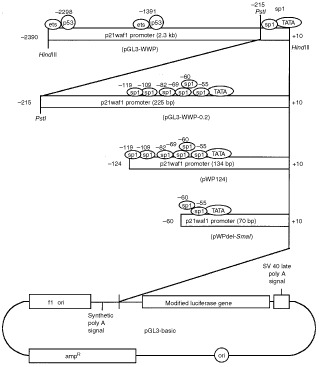
Plasmid construction. *pGL3-WWP* is a reporter construct containing 2.3 kb *p21^waf1^* promoter sequence. *pGL3-WWP-0.2*, *pWP124* and *pWPdel-SmaI* are 5′-deletion constructs of the *p21^waf1^* promoter. *pGL3-WWP-0.2* contains 225 bp of *p21^waf1^* promoter sequence. *pWP124* contains 134 bp and *pWPdel-Sma*I contains 70 bp of *p21^waf1^* promoter sequence.

The human wild-type *p21^waf1^* promoter luciferase fusion plasmid, *WWP-Luc* (El-Deiry *et al*, 1993), was a kind gift from Dr B Vogelstein (The Johns Hopkins Oncology Center). The 2.4-kilobase pair genomic fragment was subcloned into *Hind*III (Takara Biomedicals, Kusatsu, Japan) site of the luciferase reporter vector, *pGL3-Basic* (Promega, Madison, WI, USA) to generate *pGL3-WWP* (Kawamata *et al*, 1999) (Figure 1

). *pGL3-WWP* was digested with *Pst*I (Takara Biomedicals) and *Bgl*II (Takara Biomedicals), and re-ligated to generate *pGL3-WWP-0.2* (Figure 1). *pWP124* and *pWPdel-SmaI* (Nakano *et al*, 1997) (Figure 1) were kind gifts from Dr Toshiyuki Sakai (Kyoto Prefectural University of Medicine).

### Transient transfection and luciferase assay

TYS cells (5×10^5^ cells dish^−1^) were seeded in 35 mm culture dish (Falcon; Becton Dickinson Labware, Lincoln Park, NJ, USA) in DMEM supplemented with 10% FCS. Twenty-four hours later, the cells were transfected with 5 μg of reporter plasmid DNA by using Superfect reagent (QIAGEN, Hilden, Germany). Fifteen hours after transfection, vesnarinone (50 μg ml^−1^) was added, and 20 h later, cell lysates were collected. Luciferase activities were measured by Promega luciferase assay Kit (Promega). The luciferase activities were normalised by the amount of protein. Each experiment was repeated at least three times.

### Electrophoretic mobility-shift assay

**Figure 2:**
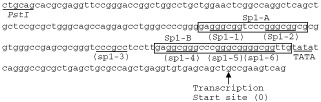
Human *p21^waf1^* promoter sequence located between −215 bp and +19 bp. The transcription start site is indicated by the number 0 on the sequence. Sp1 binding sites tentatively termed *Sp1-1, Sp1-2, Sp1-3, Sp1-4, Sp1-5* and *Sp1-6* from the upstream are indicated by underlining and shown below the sequence. *Sp1-A* contains *Sp1-1* and *Sp1-2* sites, and *Sp1-B* contains *Sp1-4, Sp1-5* and *Sp1-6* sites. TATA box is also indicated by underlining.

TYS cells (1.5×10^6^ cells dish^−1^) were seeded in 100 mm culture dish (Falcon) in DMEM supplemented with 10% FCS. Twenty-four hours later, cells were treated with vesnarinone (50 μg ml^−1^) for 15, 30 and 45 min. Cell lysates were prepared according to the method described by Chin *et al* (1996). In brief, cells were lysed with 50 mM HEPES-KOH (pH 7.9) buffer containing 400 mM NaCl, 0.2% NP-40, 10% glycerol, 0.1 mM EDTA, 1 mM dithiothreitol (DTT), 1 mM sodium orthovanadate, 0.5 mM phenylmethanesulphonyl fluoride (PMSF), 1 μg ml^−1^ of aprotinin, 1 μg ml^−1^ of leupeptin, and 1 μg ml^−1^ of pepstatin A. The protein concentrations of samples were determined with a Bio-Rad protein assay kit (Bio-Rad, Hercules, CA, USA). Double stranded oligonucleotides, (*Sp1-A*: 5′-GAG GGC GGT CCC GGG CGG CG-3′, and *Sp1-B*: 5′-GAG GCG GGC CCG GGC GGG GCG GTT G-3′) (Figure 2

) were labelled with [γ-^32^p]ATP (Amersham Pharmacia Biotech., Uppsala, Sweden) by T4 polynucleotide kinase (Promega), and purified by a spin column system (Amersham Pharmacia Biotech.). *Sp1-A* contains two Sp1 sites, and *Sp1-B* contains three Sp1 sites (Figure 2). The binding reaction mixtures consisted of 12 μg of cell lysates and 1 μl of the radiolabelled probe (approximately 5×10^4^ c.p.m.) in a binding buffer of 10 mM HEPES-KOH (pH 7.9), 0.1 mM EDTA, 0.01% NP-40, 100 μg ml^−1^ of poly (dI-dC) (Amersham Pharmacia Biotech.), and 5% glycerol. The reaction was allowed to proceed for 20 min at room temperature before loading on 6% polyacrylamide gel at a low-ionic-strength buffer (0.5×TBE). The gels were run at 100 V on ice for approximately 1 h and dried. The dried gels were exposed to X-ray film. For supershift experiments, anti-Sp1 and/or anti-Sp3 antibody (Santa Cruz Biotechnology, Santa Cruz, CA, USA) was added to the reaction mixture, and the mixture was incubated for 20 min at room temperature before addition of the radiolabelled oligonucleotide.

### Histone acetylation in TYS cells by vesnarinone treatment

TYS cells were seeded in 100 mm culture dishes. Twenty-four hours later, vesnarinone (50 μg ml^−1^) or TSA (10 μg ml^−1^) was added to the medium. Sixteen hours later, the cells were collected and the nuclear extracts were prepared as follows; cells were suspended in 400 μl of hypotonic buffer (20 mM HEPES-KOH (pH 7.9) containing 1 mM EDTA, 1 mM DTT, 20 mM NaF, 1 mM sodium orthovanadate, 0.5 mM PMSF, 0.2% NP-40, 1 μg ml^−1^ leupeptin, 10 units ml^−1^ aprotinin, and 1 μg ml^−1^ pepstatin A). Samples were centrifuged at 15 000 r.p.m. and the pellets were resuspended in 200 μl of hypertonic buffer (20 mM HEPES-KOH (pH 7.9) containing 1 mM EDTA, 1 mM DTT, 20 mM NaF, 1 mM sodium orthovanadate, 0.5 mM PMSF, 0.2% NP-40, 420 mM NaCl, 20% glycerol, 1 μg ml^−1^ leupeptin, 10 units ml^−1^ aprotinin, and 1 μg ml^−1^ pepstatin A). Samples were incubated on ice for 20 min and were centrifuged at 15 000 r.p.m. for 15 min. The supernatants were used as nuclear extracts. The protein concentrations of samples were determined with a Bio-Rad protein assay. Samples were electrophoresed on SDS-polyacrylamide gel. Proteins from gels were transferred to nitrocellulose (Bio-Rad) and were detected with an anti-acetylated Histone H3 antibody (Upstate Biotechnology, Lake Placid, NY, USA) and an Amersham ECL kit (Amersham Pharmacia Biotech.).

## RESULTS

### Effect of vesnarinone on the activation of* p21^waf1^* promoter

**Figure 3:**
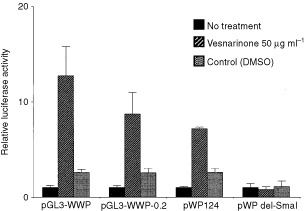
Luciferase assay. TYS cells were seeded in 35 mm dishes in DMEM supplemented with 10% FCS. Twenty-four hours later, the cells were transfected in triplicate with 5 μg of the several reporter plasmids by use of the Superfect reagent. Fifteen hours after transfection, vesnarinone (50 μg ml^−1^) was added, and 20 h later, cell lysates were collected. The luciferase activities of the cell lysates were measured with a Promega luciferase assay kit. Luciferase activities were normalized by the amount of protein in cell lysates. Data are shown as means (bars, s.d.), and are representative of three separate experiments with similar results.

Several reporter plasmids (Figure 1) were transiently transfected in TYS cells, and luciferase activity was examined. Vesnarinone apparently enhanced the luciferase activity from the *pGL3-WWP* reporter plasmid in TYS cells when compared with untreated control or DMSO treatment (Figure 3

). Vesnarinone also enhanced the luciferase activity from the *pGL3-WWP-0.2* plasmid, which contained a 215 bp promoter fragment lacking two p53 binding sites (Figure 3). Surprisingly, vesnarinone also enhanced the luciferase activity from the *pWP124* containing only a 124 bp promoter fragment. However, vesnarinone did not activate a 60 bp promoter fragment of *p21^waf1^* in *pWPdel-Sma*I reporter plasmid (Figure 3).

### Electrophoretic mobility-shift assay

**Figure 4:**
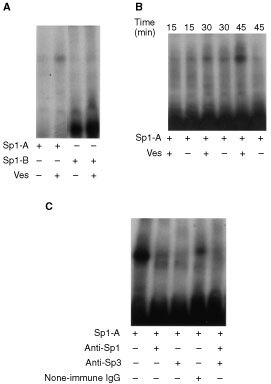
Electrophoretic mobility-shift assay (**A**, **B**) and supershift assay (**C**). Nuclear extracts prepared from vesnarinone (50 μg ml^−1^)- or DMSO-treated TYS cells were incubated with a ^32^P-labelled *Sp1-A* probe or *Sp1-B* probe (**A**). Nuclear extracts from TYS cells after treatment with vesnarinone for 15, 30, 45 min and a labelled *Sp1-A* probe were incubated in the binding buffer (**B**). Protein samples were prepared from TYS cells after treatment with vesnarinone for 45 min. Polyclonal antibody against Sp1 and/or Sp3 was added to the binding reaction and incubated for 20 min at room temperature before addition of a labelled *Sp1-A* probe (**C**).

According to the results from the luciferase assay, the vesnarinone-responsive element exists within 77 bp region relative to the TATA element. This 77 bp region harbours four independent and two overlapping nearly consensus binding sites for transcription factor Sp1. They are tentatively termed *Sp1-1, Sp1-2, Sp1-3, Sp1-4, Sp1-5* and *Sp1-6* from the upstream (Figure 2). To determine if Sp1 or other proteins can interact with the vesnarinone-responsive element, electrophoretic mobility-shift assay was performed using the oligonucleotides containing the Sp1-binding sites. The *Sp1-A* contains *Sp1-1* and *Sp1-2* sites, and the *Sp1-B* contains *Sp1-4, Sp1-5* and *Sp1-6* sites (Figure 2). After treatment with vesnarinone for 30 min, we detected the shifted band when using the *Sp1-A* as a probe (Figure 4A

). However, when we used the *Sp1-B*, we could not detect any shifted bands after vesnarinone treatment (Figure 4A). As shown in Figure 4B, the mobility shift was detectable at 30 min after treatment with 50 μg ml^−1^ vesnarinone, and the intensity of the shifted band increased at 45 min after treatment. Moreover, this band completely disappeared by adding excess unlabelled *Sp1-A* oligonucleotide (data not shown).

### Supershift assay

To elucidate whether the retarded bands represent the binding of Sp1 or Sp3, supershift assay was performed by the nuclear extracts pre-incubated with anti-Sp1 or anti-Sp3 antibody (Figure 4C). In the presence of anti-Sp1 or anti-Sp3 antibody, the intensity of the shifted band was markedly reduced. The shifted band completely disappeared in the presence of anti-Sp1 and anti-Sp3 antibody together. When pre-immune rabbit-IgG was added as a negative control, the intensity of the band was slightly reduced. However, the effect of pre-immune IgG was much weaker than that of anti-Sp1 or anti-Sp3 antibody. Thus, the effect of pre-immune IgG was probably due to non-specific interference of the IgG protein with the binding of Sp1 or Sp3 protein and DNA.

### Histone acetylation in TYS cells induced by vesnarinone

**Figure 5:**
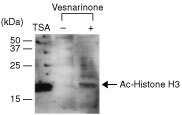
Histone acetylation in TYS cells induced by vesnarinone. Nuclear extracts were prepared from TYS cells after treatment with 50 μg ml^−1^ vesnarinone or 10 μg ml^−1^ TSA for 16 h. Protein samples were subjected to SDS–PAGE, transferred to nitrocellulose, and detected with an anti-acetylated Histone H3 antibody and Amersham ECL kit.

We investigated whether or not vesnarinone induced histone acetylation in TYS cells. Vesnarinone clearly induced histone acetylation in TYS cells like as TSA did (Figure 5

).

## DISCUSSION

In this study, we examined the molecular mechanisms of the transcriptional regulation of *p21^waf1^* gene by a differentiation inducing drug, vesnarinone. We identified the minimal vesnarinone-responsive element in the *p21^waf1^* promoter at −124 to −61 relative to the transcription start site, and demonstrated that vesnarinone enhanced the binding of the transcription factors Sp1 and Sp3 to the vesnarinone-responsive element. Furthermore, we found that vesnarinone induced the histone acetylation in TYS cells.

Sp1 is a ubiquitously expressed nuclear protein that is initially identified as a protein that binds and stimulates transcription of simian virus 40 early promoter (Dynan and Tjian, 1983). Sp1 protein binds to the GC-rich sequences present in a variety of cellular and viral promoters and stimulates their transcriptional activity (Lania *et al*, 1997). Sp3 belongs to the same family of Sp1 related transcription factor, and it also binds to the GC-rich sequences (Sp1 binding sites) (Lania *et al*, 1997). In the *p21^waf1^* promoter, there are four independent Sp1 binding sites (*Sp1-1*–*Sp1-4*) and two overlapping Sp1 binding sites (*Sp1-5, Sp1-6*) (Figure 2). We identified the *Sp1-1* and *Sp1-2* site as main vesnarinone-responsive elements of the *p21^waf1^* promoter. Generally, eukaryotic transcription is regulated by more than one transcription factor, and these transcription factors form a complex in specific promoter elements via interaction with various cofactors (Struhl and Moqtaderi, 1998). Sp1 and Sp3 are likely to be transcription factors that have low specificity to the extra-cellular stimuli, but they would be indispensable factors in p53-independent pathway on the *p21^waf1^* gene transcriptional activity in our system.

Vesnarinone induced histone acetylation in TYS cells. Recent studies demonstrated that there were various kinds of histone acetyltransferase (HAT) and histone deacetylase (HDAC) in mammalian cells, and the level of histone acetylation was controlled by equilibrium of the activities of HAT and HDAC (Grunstein, 1997). The transcriptional coactivators, p300 and CREB binding protein (CBP) are known to possess the HAT activity, and interact with a wide range of DNA binding proteins, including Sp1, p53, the RelA (p65) nuclear factor κB subunit, E2F, MyoD, activator protein 1, several nuclear receptors, and many others (Yuan *et al*, 1996; Avantaggiati *et al*, 1997; Gu and Roeder, 1997; Lee *et al*, 1998; Ikeda *et al*, 2000). Although data was not shown, we confirmed the expressions of p300, CBP and HDAC1 proteins in the nucleus of TYS cells.

Several histone acetylation inducing drugs show the growth-inhibitory effect or differentiation-inducing effect, and are used as a chemotherapeutic agent on several human malignancies (Chen *et al*, 1997; Dion *et al*, 1997; Mccaffrey *et al*, 1997). The molecular targets for the differentiation inducing drugs (or histone acetylation inducing drug) may be different from those for DNA-damaging drugs. Moreover, activating pathway of the target molecules by differentiation inducing drugs may also be different from those by DNA-damaging drugs. Thus, the differentiation inducing drugs, such as vesnarinone may act synergistically on the induction of *p21^waf1^* gene with the DNA-damaging therapy, such as radiation and the administration of conventional chemotherapeutic drugs. These informations are useful for creating new strategy for differentiation-inducing-therapy.
